# Supplementary material for: Assessing gut microbial provisioning of essential amino acids to host in a mouse model with reconstituted gut microbiomes
Source: Commun Biol. 2025 Nov 18;8:1604. doi: 10.1038/s42003-025-08966-0 (PMC12627464; doi:10.1038/s42003-025-08966-0)
Supplement: Supplementary file 3 — Description of Additional Supplementary Materials [file 42003_2025_8966_MOESM3_ESM.pdf]

Description of Additional Supplementary Files

File name: Supplementary Data 1

Description: The source data behind the graph in Figure 6

File name: Supplementary Data 2

Description: The source data behind the graph in Supplementary Figure 1
