# Supplementary material for: Assessing gut microbial provisioning of essential amino acids to host in a mouse model with reconstituted gut microbiomes
Source: Commun Biol. 2025 Nov 18;8:1604. doi: 10.1038/s42003-025-08966-0 (PMC12627464; doi:10.1038/s42003-025-08966-0)
Supplement: Supplementary file 6 — Reporting Summary [file 42003_2025_8966_MOESM6_ESM.pdf]

Corresponding author(s): *Double-anonymous peer review submissions: write DAPR and your manuscript number here instead of author names.*

Last updated by author(s): *YYYY-MM-DD*

Reporting Summary

Nature Portfolio wishes to improve the reproducibility of the work that we publish. This form provides structure for consistency and transparency in reporting. For further information on Nature Portfolio policies, see our Editorial Policies and the Editorial Policy Checklist.

Statistics

For all statistical analyses, confirm that the following items are present in the figure legend, table legend, main text, or Methods section.

n/a	Confirmed	
☐	☒	The exact sample size (n) for each experimental group/condition, given as a discrete number and unit of measurement
☐	☒	A statement on whether measurements were taken from distinct samples or whether the same sample was measured repeatedly
☐	☒	The statistical test(s) used AND whether they are one- or two-sided<br>*Only common tests should be described solely by name; describe more complex techniques in the Methods section.*
☒	☐	A description of all covariates tested
☐	☒	A description of any assumptions or corrections, such as tests of normality and adjustment for multiple comparisons
☐	☒	A full description of the statistical parameters including central tendency (e.g. means) or other basic estimates (e.g. regression coefficient) AND variation (e.g. standard deviation) or associated estimates of uncertainty (e.g. confidence intervals)
☐	☒	For null hypothesis testing, the test statistic (e.g. F, t, r) with confidence intervals, effect sizes, degrees of freedom and P value noted<br>*Give P values as exact values whenever suitable.*
☒	☐	For Bayesian analysis, information on the choice of priors and Markov chain Monte Carlo settings
☒	☐	For hierarchical and complex designs, identification of the appropriate level for tests and full reporting of outcomes
☒	☐	Estimates of effect sizes (e.g. Cohen's d, Pearson's r), indicating how they were calculated

Our web collection on statistics for biologists contains articles on many of the points above.

Software and code

Policy information about availability of computer code

Data collection	No custom or commercial software was used for data collection. Stable isotope analyses were performed at the University of California, Davis Stable Isotope Facility using standard instrument software for GC-IRMS operation (Thermo Trace GC 1310 and Delta V Advantage), as per facility protocols (lines 304–306)
Data analysis	All statistical analyses and microbiome data processing were conducted in R (version 4.2.2; http://www.R-project.org), using the following packages:<br><br>DADA2 (v1.22) for amplicon sequence processing<br>MASS for linear discriminant analysis<br>Phyloseq for microbiome data handling<br>MicrobiomeAnalyst (web platform) for comparative analyses<br>JMP Pro 17 (SAS Institute) for visualization (relative abundance plots)<br>Custom scripts and code for amplicon processing and statistical analyses are available at: https://github.com/gcuster1991/ayayee_mouse_2023.git (lines 335–336, 366–368)

For manuscripts utilizing custom algorithms or software that are central to the research but not yet described in published literature, software must be made available to editors and reviewers. We strongly encourage code deposition in a community repository (e.g. GitHub). See the Nature Portfolio guidelines for submitting code & software for further information.

Data

Policy information about availability of data

All manuscripts must include a data availability statement. This statement should provide the following information, where applicable:

- Accession codes, unique identifiers, or web links for publicly available datasets
- A description of any restrictions on data availability
- For clinical datasets or third party data, please ensure that the statement adheres to our policy

Raw 16S rRNA gene sequence reads have been deposited in the NCBI Sequence Read Archive under BioProject accession number PRJNA927293. All code used for amplicon processing and statistical analyses is available at https://github.com/gcuster1991/ayayee_mouse_2023.git. Source data for all figures and tables, including δ^{13}C-EAA values and microbiome abundances, are provided in Supplementary Tables S2 and S3. There are no restrictions on data availability.

Research involving human participants, their data, or biological material

Policy information about studies with human participants or human data. See also policy information about sex, gender (identity/presentation), and sexual orientation and race, ethnicity and racism.

Reporting on sex and gender	NA
Reporting on race, ethnicity, or other socially relevant groupings	NA
Population characteristics	NA
Recruitment	NA
Ethics oversight	NA

Note that full information on the approval of the study protocol must also be provided in the manuscript.

Field-specific reporting

Please select the one below that is the best fit for your research. If you are not sure, read the appropriate sections before making your selection.

☒ Life sciences ☐ Behavioural & social sciences ☐ Ecological, evolutionary & environmental sciences

For a reference copy of the document with all sections, see nature.com/documents/nr-reporting-summary-flat.pdf

Life sciences study design

All studies must disclose on these points even when the disclosure is negative.

Sample size	Ten germ-free (GF) C57BL/6 mice were used; five were conventionalized (CVZ) via FMT, and five remained GF (lines 257–261, 624–625).<br>Sample size was based on cost and minimum statistical relevance for isotope and microbiome studies (lines 277–278).
Data exclusions	No data were excluded from the main isotope analyses.<br>For microbiome sequencing, GF samples were removed after confirming germ-free status due to low or absent ASVs (lines 128–130, 129–130).
Replication	Each animal was an independent biological replicate; all measurements (isotope and microbiome) were performed on distinct animals (lines 257–284, 624–627).<br>All attempts at replication were successful for the main outcomes
Randomization	Mice were allocated to groups based on their gnotobiotic status (GF or CVZ) rather than by randomization (lines 260–261).<br>Randomization was not relevant due to the nature of the experimental design (pre-existing GF status).
Blinding	Investigators were not blinded during group allocation or data analysis (blinding is not relevant as groups were objectively defined by microbiological status).

Reporting for specific materials, systems and methods

We require information from authors about some types of materials, experimental systems and methods used in many studies. Here, indicate whether each material, system or method listed is relevant to your study. If you are not sure if a list item applies to your research, read the appropriate section before selecting a response.

Materials & experimental systems

n/a	Involved in the study
☒	☐ Antibodies
☒	☐ Eukaryotic cell lines
☒	☐ Palaeontology and archaeology
☐	☒ Animals and other organisms
☒	☐ Clinical data
☒	☐ Dual use research of concern
☒	☐ Plants

Methods

n/a	Involved in the study
☒	☐ ChIP-seq
☒	☐ Flow cytometry
☒	☐ MRI-based neuroimaging

Animals and other research organisms

Policy information about studies involving animals; ARRIVE guidelines recommended for reporting animal research, and Sex and Gender in Research

Laboratory animals	C57BL/6 (B6) mice, germ-free at study onset, n = 10 (5 GF, 5 CVZ), housed in flexible film isolators or individually ventilated cages under gnotobiotic conditions (Nebraska Gnotobiotic Mouse Program). See Methods lines 257–261, 624–625
Wild animals	NA
Reporting on sex	Sex was not considered in the study design and was not recorded; all analyses were performed irrespective of sex.
Field-collected samples	NA
Ethics oversight	All animal procedures were approved by the Institutional Animal Care and Use Committee at the University of Nebraska-Lincoln (protocol #2126) (lines 364–365)

Note that full information on the approval of the study protocol must also be provided in the manuscript.

Plants

Seed stocks	NA
Novel plant genotypes	NA
Authentication	NA
